# Perception of Occupational and Environmental Risks and Hazards among Mineworkers: A Psychometric Paradigm Approach

**DOI:** 10.3390/ijerph19063371

**Published:** 2022-03-12

**Authors:** Mahmaod Alrawad, Abdalwali Lutfi, Sundus Alyatama, Ibrahim A. Elshaer, Mohammed Amin Almaiah

**Affiliations:** 1 Quantitative Method Department, College of Business Administration, King Faisal University, Al Ahsa 31982, Saudi Arabia; malrawad@kfu.edu.sa; 2 Department of Accounting, Finance and Banking, College of Business Administration and Economics, Al-Hussein Bin Talal University, Ma’an 71111, Jordan; 3 Accounting Department, College of Business Administration, King Faisal University, Al Ahsa 31982, Saudi Arabia; 4 College of Business Studies, The Public Authority for Applied Education, Kuwait 13092, Kuwait; sk.alyatama@paaet.edu.kw; 5 Management Department, College of Business Administration, King Faisal University, Al Ahsa 31982, Saudi Arabia; ielshaer@kfu.edu.sa; 6 Faculty of Tourism and Hotel Management, Suez Canal University, Ismailia 41522, Egypt; 7 Department of Computer Networks, College of Computer Sciences and Information Technology, King Faisal University, Al Ahsa 31982, Saudi Arabia; malmaiah@kfu.edu.sa

**Keywords:** risk perceptions, safety and health, safety culture, psychometric paradigm, risk communication, occupational and environmental risks

## Abstract

This study aims to assess workers’ perception of occupational and environmental risks and hazards using the psychometric paradigm. For this purpose, data were collected using survey questionnaires from 360 mineworkers recruited from mineral and sand mines. Respondents were asked to evaluate eight occupational and environmental risks and hazards on nine commonly used risk characteristics. The principal component analysis revealed that two components, “Dreaded” and “Unknown”, explained 73% percent of the total variance in workers’ risk perception. The results also showed that the risk of developing an occupational disease was perceived as the most dreaded and unknown type of risk, while landslide, occupational noise, and vibration exposure were the least familiar to the respondents. A practical implication of this research is that the results may offer an insight into the employees’ perceptions of the hazards and risks associated with their working environment. This could help risk management develop and implement effective risk management and communications strategies.

## 1. Introduction

Working in the mining and quarrying sector is often considered one of the most dangerous occupations [1,2]. The International Labour Organization states that worldwide, more than two million fatal accidents occur annually; 8% are mining-related accidents, even though the mining workforce represents only 1% of the global workforce. Hence, law regulation, health, and safety worldwide have additionally pressurized the mining industry to develop and deploy new strategies to reduce accidents and fatalities [3]. In Europe, for instance, the code of practice includes risk management and assessment as mandatory requirements for plant safety and storage of hazardous chemicals [4]. This trend has directed the mining industry to prioritize occupational health and safety and to comprise various forms of risk assessment and management systems in their operations.

However, the complete integration of these systems into the business processes is challenging, particularly regarding allocating resources and staff, providing training programs, and, more importantly, developing and maintaining a robust risk culture inside the organization. For instance, positive risk culture is considered crucial in determining a risk management system’s success [5]. However, building this culture requires the organization to understand how employees view and perceive various risks and hazards associated with the working environment [6,7]. Workers’ risk perception is also essential in building an effective risk communication strategy that meets the organization’s requirements [8,9]. Therefore, assessing workers’ perceptions and understanding what forms their perceptions will enable risk management to design and implement effective strategies to eliminate or reduce the negative impact of these risks and hazards [8,10].

The vital role that risk perception plays in risk management success is well documented in the literature [7,8,11,12,13,14]. Nonetheless, much of the research related to occupational and environmental risk perception has been conducted using assessment methods designed to measure experts’ assessment (e.g., Analytical Hierarchical Process, cost–benefit analysis, and severity–frequency analysis) [7,8,11,12,13]. However, these methods should be used mainly with risk experts who have sufficient experience and knowledge about risk assessment processes. On the other hand, non-expert workers tend to view risks differently; therefore, their perception should be measured using non-expert methods such as the psychometric paradigm. Accordingly, the present research aims to bridge the gap in the literature by proposing and empirically testing the psychometric paradigm’s effectiveness in measuring workers’ perception of occupational and environmental risk and hazards.

The remainder of this paper has been organized as follows. Section 2 summarizes the literature related to current occupational health and safety strategies and the psychometric paradigm. Section 3 discusses the materials and methods used in this study, including the questionnaire development. Furthermore, Section 4 presents a summary of the study results a discussion. Finally, Section 5 discusses the findings and limitations and the conclusions.

## 2. The Psychometric Paradigm

Slovic and his colleagues developed the psychometric paradigm during the 1970s and the 1980s to unveil why laypeople assess the risk associated with various technologies, products, and activities differently from experts [15,16,17,18]. The model suggested that the latter build their risk assessment by quantifying uncertain events using probability and outcome values or loss severity. However, the former tends to hold subjective judgments of risk; therefore, their risk assessment depends on factors mainly related to attitudes and beliefs, such as psychological, social, institutional, and cultural aspects. Furthermore, the model proposed nine characteristics that influence an individual’s perception: individual knowledge, science knowledge, newness, commonness, control, immediacy, chronic, voluntary, and severity [15,17]. These were also assumed to capture the multidimensionality of individual risk perceptions. Appendix A, Table A1 shows the list of attributes used to measure risk perception in this study, with a description of the scale employed [19].

Briefly, in the psychometric approach, individuals are requested to rate their perception of various risks and hazards based on several common risk characteristics identified in previous studies [20,21,22,23,24,25]. Subsequently, a principal component analysis (PCA) was used to reduce the number of proposed characteristics into two main dimensions or factors, frequently identified and named as “dreaded” and “unknown risks.” The former refers to the extent to which a threat is perceived as being dreadful, uncontrollable, and involuntary, with a catastrophic potential and fatal consequences; however, the latter entails the extent to which an event or hazard is believed to be unobservable, unknown to individuals, unfamiliar, new, and has delayed consequences [15,17].

Many prior studies have identified the aforementioned two main dimensions [22]. However, other research, such as Bronfman et al. [20], identified four dimensions of risk and labeled them as catastrophic potential, knowledge, involuntariness, and social and personal exposure. The first dimension, “catastrophic potential” is related to characteristics such as “Dread” “Severity of the consequence” and “Catastrophic potential.” The second dimension, “knowledge” is associated with “Knowledge” “Oldness” and “Latency of effect.” Moreover, the third dimension, “involuntariness” encompasses involuntariness and control. The last component, “social and personal exposure” relates to “Number of people exposed” and “Personal effect.” The psychometric approach has been widely used to assess laypeople’s risk perception of various risks and hazards, including food processing technologies [10,21,26], social risk [27,28], nuclear energy technologies [29,30], environmental, natural, and climate change-related risks [31,32,33], and industrial accidents [34].

Considerable occupational and environmental risk perception research has been conducted using various risk assessment methods, such as the analytical hierarchical process (AHP), cost–benefit analysis, checklist, and severity–frequency analysis [7,8,11,12,13]. These methods were initially designed to examine expert perceptions of risks and hazards. However, insufficient studies have attempted to understand workers’ risk perception using non-expert risk assessment approaches such as the psychometric paradigm. The present study extended the recent research on occupational and environmental risk perception by evaluating mineworkers’ risk perception using the psychometric paradigm. Furthermore, it included a list of hazards that had never been the subject of prior psychometric research, including respiratory-related issues, occupational noise and vibration exposure, risk of developing occupational diseases, and equipment accidents.

Understanding risk perception is essential for developing and implementing effective communication and management strategies [35]. Measuring workers’ risk perception regarding various work activities and identifying the characteristics that drive it will enable the risk management to develop and implement effective risk communication strategies that meet the organization’s requirements and address workers’ worries [36,37]. Furthermore, this will help the risk management to focus their efforts and resources on building and sustaining a positive risk culture.

## 3. Materials and Methods

### 3.1. Study Design

The present study design follows the tradition of earlier psychometric paradigm research. First, a literature review related to the psychometric paradigm was conducted. Second, a list of related risks and hazards is formulated. Third, a survey questionnaire was developed based on the literature and the risks and hazards identified in the second step. Finally, analyzing the collected data using several statistical techniques and ending with the discussion and conclusion. The remainder of this section will describe the development of the research measurement, the study population and data collection, and the statistical methods used in the analysis.

### 3.2. Measurement Development

The data were collected using a survey questionnaire developed by the authors based on previous literature [38,39]. It consisted of two sections. The first one aimed to gather demographic information, including age, gender, experience, and type of work. The second section purported to measure individuals’ risk perception regarding the pre-selected list of risks and hazards listed (including: Risk of developing an occupational disease, Occupational noise and vibration exposure, Dust exposure, Respiratory-related issues caused, exposure to harmful substances, Landslide, Equipment accidents, and Explosive) using the nine risk characteristics, including knowledge of exposure, knowledge of science, newness, common/dreaded, control, immediacy, chronic/catastrophic, voluntariness, and severity of the risk. These risk characteristics found in Appendix A have been commonly used in prior psychometric studies [20,28,40,41,42]. The respondents were requested to assess these risks using a seven-point Likert scale ranging from one (low) to seven (high), as shown in Appendix A, Table A1. Before proceeding with data collection, ethical approval was sought and granted for the developed questionnaire from the research ethics committee at Al Hussein Bin Talal university—Jordan (Ref No: Ra-3-10-1481, date of approval: 24 June 2018).

### 3.3. Study Population and Data Collection

The participants were recruited from two phosphate mines located in Jordan and Egypt. Eshidiya Mine is located in the southern part of Jordan, around 70 KM away from Ma’an District. The mine produces 66% of the country’s dried phosphate, and the total number of employees in this mine is around 792 [43]. The second is Abu Tartour, a phosphate mine located in Egypt’s western desert around 50 KM west of El Kharga City, the capital of the New Valley Governate in southwestern Egypt. The total number of employees is around 3750. Accordingly, the population of this study consists of 4542 employees from both mines. The inclusion criteria used in this study included participation working on the main site of the mine. The only exception criteria were related to the participant’s literacy skills and willingness to participate in the study.

Four hundred and eighty questionnaires were distributed using simple random sampling techniques with the help of two research assistants from each country. Overall, 360 participants’ responses were received, with a response rate of 75%. Table 1 shows the sample’s sociodemographic profile: 63% (*n* = 225) and 37% (*n* = 135) were from Egypt and Jordan, respectively. Furthermore, 53% (*n* = 192), 44% (*n* = 158), and only 3% (*n* = 10) were in their 30 s and below, 40 s, and 50 s and above, respectively. The profile also showed that 94% (*n* = 337) of the respondents were non-managerial employees, and only 6% (*n* = 23) were managerial. Furthermore, information regarding their previous experience demonstrated that 87% (*n* = 315) and merely 13% (*n* = 45) had less and more than five years of experience, respectively.

### 3.4. Methods of Data Analysis

In order to measure workers’ perception of occupational and environmental risks and hazards, this study used three statistical techniques, including principal component factor analysis, multiple regression analysis, and Tukey’s post-hoc test. First, following the introduction of the psychometric analysis, the principal component factor analysis with varimax rotation was used on the aggregated data to reduce risk characteristics and identify the most relevant risk factors. Secondly, a stepwise multiple regression analysis was conducted using the disaggregated data to examine if the qualitative risk characteristics can predict workers’ perception of the selected list of risks and hazards and develop the heat map for the selected list of risks. Finally, Tukey’s post-hoc test was conducted to determine if the differences in the overall risk perception for the list of risks and hazards are statistically significant. All of the techniques mentioned above were performed using IBM SPSS 23.

## 4. Results

### 4.1. Data Preparation

Before the analyses, the data were screened for outliers and missing values. The outlier screen and unengaged respondents were examined using the standard deviation for each respondent’s survey item [39]. A low or zero standard deviation indicated that the respondents entered the same pattern (e.g., 1,1,1,1, or 5,5,5,5), indicating that they were not engaged and their responses were eliminated. For missing values, Fife-Schaw and Rowe [44] recommended that hazards with over 15% missing values be excluded from the analysis to ensure the results’ accuracy. Accordingly, 15 cases were dismissed from the study. However, those responses with less than 15% missing data were treated by replacing the missing values with the mean ones, as Jenkins et al. [20] suggested. Subsequently, the data were analyzed using the method outlined in [20,21,44,45]. The screening process has brought the final sample to 345 valid responses.

### 4.2. Aggregate-Level Hazard-Focused Analyses

First, the data were averaged for all responses to produce a mean rating for all eight hazards for each of the nine risk characteristics (for example, Characteristics 1− Risk1 + Char 1− R2 + Char 1−R3 +….+ Char 1− R9). Subsequently, the aggregated data were subjected to a PCA with varimax rotation to extract the two components suggested by the psychometric literature. The data’s suitability for the PCA was tested using the Kaiser-Meyer-Olkin (KMO) and Bartlett’s test of sphericity. As shown in Table 2, the KMO (=0.78) test indicated that the factor analysis was appropriate for the current data. Moreover, Bartlett’s test of sphericity (*χ*^2^ (21) = 511.77, *p* < 0.001) supported the data suitability [39,46].

The PCA revealed that seven out of nine risk characteristics were loaded onto two factors, while two risk characteristics (severity of risk and knowledge of science) had cross-loading issues. In addition, their communalities were lower than the cutoff value of 0.05, as recommended by Hair [46]. Therefore, both were excluded from further analysis. The reliability test showed that Cronbach’s alpha coefficient for both factors (Factor 1 = 0.88 and Factor 2 = 0.77) exceeded 0.60, the cutoff value recommended by social science researchers [46].

The resulting two factors explained 73.5% of the total variance in the characteristic rating. Table 3 presents the orthogonal components of the rotated factor loading. The first factor accounted for 43.80% of the total variance and was labeled “dreaded risk.” Four of the seven risk characteristics were loaded on this factor (control, command/dreaded, immediacy, and chronic/catastrophic). The second factor accounted for 29.7% of the variance and was labeled as an “unknown risk.” Three of the seven risk characteristics were loaded on this factor: newness, knowledge of exposure, and voluntariness. The reliability of the PCA solution was tested using Cronbach’s alpha.

The PCA results and the aggregated data were used to build a cognitive map of the selected list of risks. Following the procedure in [47], two-factor scores were calculated for each type of risk by weighting the ratings on each risk dimension proportionally to the significance of the factor dimension and adding the total dimensions. For example, the value for Risk 1 related to the dreaded dimension would be calculated by summating all the correlated risk characteristics (Risk 1 score = 0.91 common/dreaded + 0.85 immediacy + 0.82 control + 0.82 chronic/catastrophic). This weighted summing would determine whether an individual’s risk is high or low based on whether the ratings on the dimensions most closely linked with each of the two variables are high or low [20].

**Figure 1:**
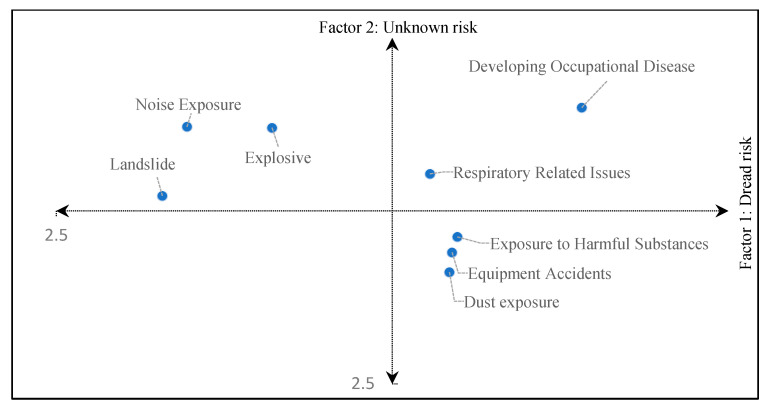
Location of risks in the plane of Principal Components 1 and 2 for the study sample.

According to Slovic [48], individual perception represents the location of a hazard within a factor space. The higher the perceived risk of an event, the greater the score for the designated factor [29]. Figure 1 shows the selected list of risks mapped into space-spanning factors 1 and 2 according to their respective scores. The map represented two main risk factors, the “dreaded” and “unknown risks.” The map’s lower and upper right quadrants showed that our respondents perceived the risks of developing an occupational disease, respiratory-related issues, exposure to harmful substances, equipment accidents, and dust exposure. The map’s upper left and right quadrants included the risks of developing an occupational disease, occupational noise and vibration exposure, landslides, explosives, and respiratory-related issues. These risks and hazards were perceived as unknown by the respondent sample.

### 4.3. Disaggregate-Level Hazard-Focused Analyses

The second part of the analysis was conducted using disaggregate-level or hazard-focused analyses. Hence, a series of eight multiple regression analyses using non-aggregated data were conducted to test if the selected qualitative risk characteristics were significant predictors of the participants’ perceived risk [21,27]. Table 4 summarizes the multiple regression analysis results. Furthermore, the table demonstrated a risk heat map for the study respondents. As revealed by the health map, three risk characteristics were the most significant predictors of the employees’ risk perception: immediacy, catastrophic, and dreaded. These three predictors explained more than 35% of the respondents’ perceptions of variance.

### 4.4. Overall Mean Risk Ratings

**Figure 2:**
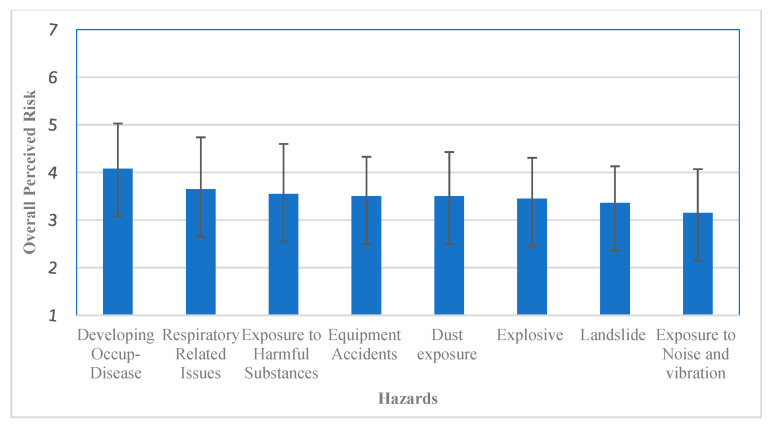
The overall mean risk ratings for each hazard. (Error bars represent ±1 standard deviation (SD)).

A one-way analysis of variance was conducted to compare the overall ratings of the selected list of risks. The results showed a significant difference between the risk ratings at *p* < 0.05 [F (7, 1272) =13.25, *p* < 0.001]. Further, the post hoc comparisons using Tukey’s honestly significant difference test indicated that the risk of developing an occupational disease and landslide was perceived to be significantly different from the other six hazards, as shown in Table 5 and Figure 2. The mean score for all risks showed that developing an occupational disease was perceived as the highest risk (M = 4.08, SD = 0.95). In contrast, occupational noise and vibration were considered the lowest among all the hazards (M = 3.15, SD = 0.92). However, there were no significant differences between respiratory-related issues, exposure to harmful substances, dust exposure, flush flooding, occupational noise, and vibration exposure. The same results were applicable to exposure to harmful substances, dust, explosive and occupational noise, and vibration.

## 5. Discussion

The main objective was to propose and test the psychometric paradigm as a methodological approach to measure workers’ perception of occupation and environmental risks and hazards and assess workers’ risk perception in the mining sector. Accordingly, a list of eight risks and nine risk’ characteristics were assessed using data collected from 360 mineworkers. Data were analyzed following psychometric studies traditions. Following is the most prominent finding to emerge from the analysis.

With respect to the first research objective, the results of the aggregated level and hazard-focused data provide some support to the use of the psychometric paradigm literature in assessing workers’ perception. In particular, the results showed that the cognitive representation of the workers’ perceptions was structured based on two well-known factors: dreaded and unknown risks. Both factors explained 74% of the variance in the respondents’ risk judgments. Consistent with the literature, unknown risk factors were associated with risk characteristics, including newness, personal knowledge of exposure, and voluntariness. Furthermore, dreaded risk factors were related to risk characteristics, comprising common/dreaded, immediacy, control, and chronic/catastrophic [20,27,40,44].

Furthermore, considerable previous literature on the psychometric paradigm has established the vital role of knowledge in shaping individuals’ risk perceptions of various technologies and activities [21,27,35,40,44,49,50,51,52,53,54]. In line with this, our results have also supported the literature by extending psychometric research to include occupational and related risks and hazards [55]. Therefore, the disaggregate analysis provides additional information on risk perceptions. For instance, the aggregate level showed that the risk of developing an occupational disease was the highest for dreaded and unknown factors. However, a disaggregated analysis offers precise characteristics and prediction power. Moreover, using it provides vital information for the risk management team that can be used to build a heat map that helps quantify and prioritize occupation and environmental risks more relevant to their employees.

### 5.1. Research Implications

This study aimed to contribute to the occupational health and safety literature and the mining industry’s risk management practices by employing a psychometric paradigm to measure workers’ perceptions of dreaded and unknown occupational and environmental risk and hazard factors. It provides new and different insights into the problem using a survey questionnaire assessing mineworkers, which might help in the development of comprehensive strategies to form an efficient safety system.

We believe that our study makes a significant contribution to the literature because insufficient studies have attempted to understand workers’ risk perception using non-expert risk assessment approaches. Thus, this study extended the recent research on occupational and environmental risk perception by employing the psychometric paradigm. Furthermore, it included a list of hazards that have previously not been the subject of psychometric research.

Additionally, this study’s findings facilitate understanding how workers perceive the risks and hazards associated with their working environment. They provide insightful information that would enable health and safety departments to develop and implement effective risk management and communication strategies. Furthermore, this would help the risk management to focus their efforts and resources on building and sustaining a positive risk culture.

### 5.2. Limitation and Suggestions for Future Research

This study is subject to the limitations inherent to retrospective studies conducted using the psychometric paradigm. These include study population, sample size, limited list of risks and hazards, data analysis method, and types of respondents. Although this study’s findings provide insights into workers’ perceptions of occupational risks, they are limited to mining. Furthermore, the tested risks and hazards list is more relevant to this mining sector. Therefore, employees working in other sectors might experience different risks and hazards and consequently have varying preferences regarding them. Another limitation of the research design is the insufficient statistical techniques employed in this study due to the small sample size. Studies using an additionally significant sample could benefit from comparing different respondent groups (e.g., male vs. female, skilled vs. semi-skilled, managerial vs. non-managerial). However, expanding the study scope and scale in future research, such as including a new list of related risks and hazards, including respondents from other complex industries, and obtaining a bigger sample size, will help improve the reliability and validity of the findings.

## 6. Conclusions

The psychometric paradigm was initially developed to examine laypeople’s risk perception of various risks and activities, including food processing technologies, nuclear energy technologies, and climate change-related risks. Therefore, this study came as an attempt to propose and examine the suitability of this approach for studying employees’ perceptions of occupational risk. The research findings supported the application of the psychometric paradigm in the health and occupational safety fields. This high explanatory power of the model, around 73% of the variance in mineworkers’ risk perception, made it suitable to assess workers’ risk perceptions of occupational and environmental risks and hazards. Understanding how employees perceive the risk of various work activities and identifying the characteristics that drive their perceptions would enable risk managers to develop and implement efficient risk communication strategies that meet their requirements and address their worries.

## Figures and Tables

**Table 1:** Sociodemographic profile of the sample.

Profile	Category	Frequency	%
Country	Jordan	135	37%
	Egypt	225	63%
Age groups	≤30	192	53%
	31–49	158	44%
	≥50	10	3%
Type of work	Managerial employees	23	6%
	Non-managerial employees	337	94%
Experience	Less than 2 years	170	47%
	2 to 5 years	145	40%
	5 to 10 years	38	11%
	More than 10 years	7	2%

**Table 2:** The test results of the Kaiser–Meyer–Olkin test and Bartlett’s test of sphericity.

Kaiser–Meyer–Olkin and Bartlett’s Test		
Kaiser–Meyer–Olkin Measure of Sampling Adequacy		0.778
Bartlett’s Test of Sphericity	Approx. Chi-Square	511.771
	df	21
	Sig.	0.000 *

Note: * Indicate significance at the level of significance (*p* < 0.01).

**Table 3:** Principal component loadings (*N* = 345).

Psychometric Characteristics	Communalities	Varimax Rotated Component Loadings	
		F1 (Dreaded)	F2 (Unknown)
Common/Dreaded	0.82	**0.91**	−0.01
Immediacy	0.78	**0.85**	−0.04
Control	0.69	**0.82**	−0.06
Chronic/Catastrophic	0.75	**0.82**	0.27
Newness	0.75	0.25	**0.83**
Knowledge of exposure	0.74	0.05	**0.85**
Voluntariness	0.70	−0.25	**0.80**
Eigenvalues		3.07	2.08
The proportion of the explained variance	73.50	43.8	29.7
Cronbach’s alpha		0.88	0.77

Note: The rotation converges in two iterations. The factor loadings over 0.40 appear in bold.

**Table 4:** Heat map of significant predictors or overall risk ratings for each hazard.

	Dreaded	Immediacy	Control	Catastrophic	Newness	Knowledge of Exposure	Voluntariness	R^2 (%)^
Developing occupational disease	0.44 *	0.04	0.13	0.10	0.22 *	0.12	0.04	56
Respiratory-related issues caused	0.27 *	0.22 *	0.17 *	0.22 *	0.13 *	0.12	0.14 *	70
Exposure to harmful substances	0.22 *	0.24 *	0.32 *	0.04	0.11	0.20 *	0.20 *	70
Equipment accidents	0.17 *	0.22 *	0.34 *	0.20 *	−0.03	0.11	0.23 *	68
Explosive	0.02	0.39 *	0.07	0.35 *	0.13	−0.01	0.19 *	60
Dust exposure	0.02	0.41 *	0.06	0.09	0.11	0.25 *	0.07	45
Landslide	0.02	−0.02	0.22 *	0.42 *	0.18 *	0.17 *	0.21 *	63
Occupational noise and vibration exposure	0.10	0.49 *	0.16 *	0.02	0.33 *	−0.01	0.05	62
	
Level of effect				Very low	Low	Medium	High	Very high
Standardized β coefficients levels				<0.2	0.2–0.24	0.25–0.29	0.3–0.34	>0.35
color scales corresponding with the level of β coefficients								

Note: Cells with no color indicate non-significant results. * *p* < 0.05. We added level raw (level of effect). The second raw was name (as stated in note 1). The third raw shows the color scales corresponding with the level of β coefficients.

**Table 5:** Post-hoc comparison using Tukey’s honestly significant difference test.

			Tukey Groups							
	Mean	SD		B	C	D	E	F	G	H
Developing an occupational disease	4.08	0.95	A	5.73 *	7.23 *	7.88 *	8.60 *	7.83 *	12.7 *	9.77 *
Respiratory-related issues	3.65	1.09	B	1	1.50	2.14	2.87	2.10	6.93 *	4.03
Exposure to harmful substances	3.55	1.05	C		1	0.64	1.37	0.60	5.44 *	2.53
Equipment accidents	3.50	0.83	D			1	0.73	0.04	4.79 *	1.89
Dust exposure	3.50	0.93	E				1	0.77	4.07	1.17
Explosive	3.45	0.86	F					1	4.84 *	1.93
Landslide	3.36	0.77	G						1	2.90
Occupational noise and vibration exposure	3.15	0.92	H							1

Note: * Indicate significance at the level of significance (*p* < 0.05).

## Data Availability

Data are available upon request from researchers who meet the eligibility criteria. Kindly contact the first author privately through e-mail.

## Appendix A

**Table A1:** Risk Attributes.

Attribute	Description of the Scale	Low (1)	High (7)
Knowledge to exposed	To what degree is the risk associated with each activity, substance, or technology known to you?	Known	Unknown
Knowledge to science	In what magnitude this activity, substance, or technology has the potential to cause death and catastrophic destruction?	Known	Unknown
Newness of risk	Is the risk associated with each activity, substance, or technology new and non-familiar, or old and familiar?	New	Old
Common/dreaded	Is the risk related to each activity, substance, or technology a common or terrible risk?	Common	Dreaded
Control over risk	To what degree can the exposed population avoid the risk associated with each activity, substance, or technology?	Uncontrollable	Controllable
Immediacy of effect	Are the effects of the risk associated with each activity, substance, or technology immediate, or do they take place later in time?	Immediate	Delayed
Chronic/catastrophic	Is the risk associated with each activity, substance, or technology new and non-familiar, or is it chronic or catastrophic?	Chronic	Catastrophic
Voluntariness of risk	To what degree is the risk associated with each activity, substance, or technology faced voluntarily by the exposed population?	Voluntary	Involuntary
Severity of risk	How likely are the consequences fatal when the risk associated with this activity, substance, or technology appears?	Not severe	Severe

Source: [19] (p. 1273).
